# Education-Related Stress and Its Behavioral and Somatic Manifestations Among Dental Students: A Cross-Sectional Analysis of Bruxism and Temporomandibular Symptoms

**DOI:** 10.3390/healthcare14010072

**Published:** 2025-12-27

**Authors:** Merve Berika Kadıoğlu, Meyra Durmaz, Mahmut Kadıoğlu

**Affiliations:** 1 Department of Orthodontics, Faculty of Dentistry, Ankara University, 06500 Ankara, Türkiye; dtmeyradurmaz@gmail.com; 2 Independent Researcher, 06500 Ankara, Türkiye; drmahmutkadioglu@gmail.com

**Keywords:** bruxism, temporomandibular disorders (TMD), education-related stress, student well-being, mental health, dental students

## Abstract

**Background/Objectives:** Dental training is known for its demanding academic pace, early clinical exposure, and constant performance pressure. These stressors may contribute to behavioral and physical manifestations, including bruxism and temporomandibular disorder (TMD). This study aimed to better understand the multidimensional burden experienced in this educational setting by investigating the relationship between education-related stress, bruxism patterns, and temporomandibular symptoms (TMD-related symptoms) in dental students. **Methods:** A cross-sectional survey was conducted at the Ankara University Faculty of Dentistry in 2025 and completed by 287 undergraduate dental students. The questionnaire collected sociodemographic information, self-reported bruxism status, TMD-related symptoms via the Fonseca Anamnestic Index (FAI), and education-related stressors using the Dental Environment Stress (DES) scale. Descriptive statistics, group comparisons, and Spearman correlation analyses were conducted. **Results:** Bruxism was reported by 76% of students and was significantly more common among females (*p* < 0.05). Students with bruxism demonstrated higher DES (3.34 ± 0.84) and FAI (41.81 ± 20.32) scores compared with those without bruxism (*p* < 0.001). DES and FAI scores showed a significant positive correlation (r = 0.229, *p* < 0.001). Stressors related to workload, examinations, limited rest time, clinical uncertainty, patient responsibility, and financial concerns were strongly associated with bruxism, while inconsistent academic feedback emerged as a key distinguishing factor. **Conclusions:** Education-related stress is closely linked to bruxism and TMD-related symptoms among dental students. Beyond overall stress intensity, the nature of experienced stressors plays a critical role. These findings highlight the importance of supportive learning structures, targeted stress-management strategies, and curriculum-level improvements to promote student wellbeing and resilience.

## 1. Introduction

According to contemporary international classifications and consensus reports, bruxism is defined as an involuntary masticatory muscle activity occurring during sleep or wakefulness, manifested in phasic, tonic, or mixed forms. While it may involve tooth clenching or grinding, bruxism itself is conceptualized as a multifactorial motor activity with biological, psychological, and environmental determinants [1]. Its prevalence has been reported as 9% in adults, 14–20% in children, 13% in young adults aged 18–29 years, and approximately 3% in older adults [2]. Temporomandibular disorders (TMD) affect approximately 34% of the global population, and epidemiological projections indicate that this prevalence may rise to nearly 44% by 2050, emphasizing the growing public health relevance of identifying factors that contribute to TMD in young adult populations [3].

Although bruxism episodes are often brief, they may lead to considerable clinical consequences, including temporomandibular joint (TMJ) pain, hypertrophy of the masticatory muscles, temporomandibular disorders, tooth wear, dental sensitivity, headaches, and even implant failure [4].

The etiology is multifactorial, involving biological, psychological, and environmental factors [5]. Emotional stress has been consistently recognized as a major contributor [6,7]. Stress-induced activation of neuroendocrine and dopaminergic pathways has been suggested as a mechanism for both sleep and awake bruxism [8,9]. Additionally, increased activation of the sympathetic nervous system has been identified as a fundamental component of the pathophysiology of sleep bruxism. Increased autonomic arousal preceding rhythmic masticatory muscle activity further supports the biological link between stress-related hyperarousal and bruxism [10]. Moreover, psychiatric conditions such as anxiety and depression have been frequently associated with bruxism and TMD [11].

Dentistry has long been associated with occupational stress and is considered one of the most stressful healthcare professions. This stress may emerge early during dental education and/or in the transition from academic life to professional practice [12]. Dental education is widely acknowledged as a demanding and stressful process and the intensity of stress among dental students has attracted the attention of many researchers worldwide [9,13]. Dental education creates a highly stressful environment because students are required to acquire theoretical knowledge, develop clinical competence, and manage patient care under strict time constraints while also demonstrating effective interpersonal skills [14]. Consequently, dental students report higher levels of psychological stress compared to many other health professional students [15,16]. Moreover, several studies have demonstrated that stress levels in dental students increase progressively across the years of study, with a pronounced escalation during the clinical years due to greater patient responsibilities, clinical requirements, and performance pressure. This year-specific rise in stress further strengthens the rationale for examining bruxism and TMD in this population [17].

To evaluate these academic, clinical, and social stressors, the Dental Environment Stress (DES) scale has been developed and validated across different cultural contexts, including Türkiye [18,19,20].

Unlike general stress measures such as the Perceived Stress Scale (PSS) or the Depression Anxiety Stress Scale (DASS-21), the Dental Environment Stress (DES) scale directly assesses stressors specific to dental education, including difficulties in learning clinical procedures, inconsistencies in academic feedback, and lack of confidence in clinical decision-making. Although the literature consistently demonstrates an association between elevated stress levels in dental students and the prevalence of bruxism and TMD, the majority of these studies have assessed stress using general psychological instruments such as the PSS or DASS-21, and the relationship between dental education-specific stress factors and bruxism has not been adequately investigated [9,11,21].

Bruxism and TMD have been reported to frequently co-occur, and these conditions may share common psychosocial and functional determinants [6,22]. In addition, the relationship among stress, TMD, and bruxism has been suggested to be bidirectional, whereby chronic TMD pain may increase psychological stress and, in turn, sustain parafunctional muscle activity. Consideration of this reciprocal interaction contributes to a better understanding of the shared pathophysiological and psychosocial background of these conditions [23]. However, despite these advances, the majority of previous studies have either focused exclusively on the association between stress and bruxism or on the relationship between bruxism and TMD, thereby neglecting the potential combined influence of stress factors on both conditions.

Importantly, few studies have specifically addressed dental education-related stressors—such as clinical workload, examination pressure, or inconsistency in academic feedback—which may represent a distinct set of challenges compared to general life stress. Since dental training uniquely combines academic, clinical, and interpersonal demands, students may be particularly vulnerable to stress-induced parafunctional behaviors. Yet, comprehensive studies that simultaneously examine the triad of bruxism, dental education-related stress, and TMD remain scarce. Addressing this gap is essential for understanding the multifactorial etiology of bruxism in dental students and for informing preventive strategies that target both psychosocial and functional domains.

The intense academic and clinical workload faced by students in health education can affect not only emotional well-being but also behavioral and physical health parameters. Therefore, addressing the somatic and behavioral consequences of education-related stress with a holistic approach has become increasingly important. Bruxism and TMD are among the common physical and behavioral manifestations of chronic stress and can affect the well-being of health professional candidates in multiple ways. In this context, evaluating the relationship between stress levels and bruxism and TMD-related symptoms in dental students provides valuable information for developing sustainable well-being strategies in health education.

In this regard, the aim of this study was to investigate the association between bruxism, temporomandibular symptoms (TMD-related symptoms) and dental education-related stress in undergraduate dental students. By employing validated instruments—the Dental Environment Stress (DES) scale for education-specific stress and the Fonseca Anamnestic Index (FAI) for temporomandibular symptoms—this research integrates psychosocial and functional dimensions that have often been studied separately. To our knowledge, this study is among the first to systematically examine the triad of bruxism, DES, and TMD-related symptoms within the same population, providing novel insights into the behavioral and functional consequences of stress in dental education and emphasizing the importance of stress management and psychological support strategies to reduce stress-related parafunctional habits and improve students’ overall well-being.

## 2. Materials and Methods

### 2.1. Study Design and Ethical Approval

This cross-sectional descriptive study was conducted at the Faculty of Dentistry, Ankara University. Ethical approval was obtained from the Ankara University Ethics Committee (Approval Date: 13 January 2025, Approval No: 5/4). Informed consent was obtained electronically from all participants prior to enrollment.

### 2.2. Sample Size Calculation and Participants

The a priori power analysis was performed using G*Power software (version 3.1; Heinrich Heine University Düsseldorf, Düsseldorf, Germany) based on a two-independent-groups comparison (bruxism vs. non-bruxism). Assuming a medium effect size (Cohen’s d = 0.5), α = 0.05, and power = 0.80, the minimum required sample was 128 participants (approximately 64 per group). Because non-parametric tests do not have dedicated power functions in G*Power, this estimation was based on the standard two-group comparison approximation. The sample was recruited using a convenience sampling method, and data were collected through an online survey prepared via Google Forms (Google LLC, Mountain View, CA, USA).

At the time of data collection, the faculty had 880 enrolled undergraduate dental students. The questionnaire was distributed to 400 students who were actively attending theoretical courses and/or clinical training during the study period. A total of 287 students (194 females, 93 males) from all academic years (1st–5th) completed the survey and met the eligibility criteria, yielding a response rate of 71.7%.

Data collection procedures were designed to ensure anonymity and voluntary participation, thereby reducing potential response bias and enhancing the reliability of self-reported data. All participants were provided with detailed information regarding the purpose, scope, objectives, and procedures of the study. They were explicitly informed that participation was entirely voluntary and that no negative consequences would result from refusal or withdrawal at any stage.

The exclusion criteria were as follows: (i) use of antidepressants or other psychiatric medications, (ii) diagnosed neurological or systemic disorders, (iii) history of botulinum toxin injection into the masticatory muscles within the last 6 months, (iv) incomplete questionnaire response, and (v) lack of informed consent.

### 2.3. Assessments

A structured questionnaire was administered to all participants. The questionnaire consisted of four sections. The first section collected demographic and socioeconomic in-formation, including participants’ age, gender, and sociodemographic characteristics. The second section assessed the presence of bruxism using a self-reported bruxism questionnaire. The third section evaluated TMD-related symptoms with the Fonseca Anamnestic Index (FAI), while the fourth section assessed stress factors related to dental education using the Dental Environment Stress (DES) scale.

#### 2.3.1. Demographic Information Form

Data on age, gender, income level, smoking & alcohol habits, and chewing side preferences were collected.

#### 2.3.2. Bruxism Assessment

Bruxism diagnosis was graded as possible, probable, and definite according to the international consensus [24]. In line with Phuong et al., this study relied exclusively on self-reported data; therefore, only possible bruxism was identified [25]. The questionnaire items were adapted from the recommendations of the American Academy of Sleep Medicine for possible sleep bruxism (SB) and from Pintado et al. for possible awake bruxism (AB) [26,27]. The questions were as follows:

1. Are you aware, or has anyone told you, that you grind your teeth during sleep?
2. Are you aware that your teeth are worn down more than normal?
3. Upon awakening, do you experience any of the following symptoms?
  (i) Jaw fatigue, tightness, or soreness
  (ii) Awareness of clenching or oral pain
  (iii) Pain in the temples
  (iv) Difficulty in opening the mouth wide
  (v) Tension in the jaw joint and the need to move the mandible
  (vi) A “clicking” sound in the jaw joint that disappears afterward
4. Are you ever aware of grinding your teeth during the day?
5. Are you ever aware of clenching your teeth during the day?

The questionnaire aimed to capture signs and symptoms related to clenching and grinding during both daytime and nighttime. Respondents were considered to have possible SB if they answered “yes” to Question 1 and/or Question 2, or to at least one of the symptoms listed in Question 3. A “yes” response to Question 4 and/or Question 5 was considered possible AB. However, in the present study, sleep and awake bruxism were not analysed separately; participants who fulfilled any of the above criteria were classified collectively as “possible bruxers.”

Because the present study relied on self-reported bruxism, the diagnostic performance of this approach was considered in light of available validation research. Laboratory-controlled studies comparing self-report items with polysomnography (PSG) or electromyographic (EMG) recordings have consistently demonstrated moderate diagnostic accuracy, supporting their use in epidemiological studies aimed at identifying possible bruxism rather than establishing a clinical diagnosis [28,29,30]. Previous investigations reported sensitivity values ranging from approximately 55–67% and specificity from 68–89%, with overall accuracy around 62–65%, indicating a reasonable but imperfect ability to distinguish bruxers from non-bruxers. In addition, meta-analytic evidence suggests that self-report tools may underestimate true bruxism prevalence, particularly for sleep bruxism, while maintaining acceptable specificity—an expected limitation of non-instrumental diagnostic methods [31]. Accordingly, although self-report measures cannot classify probable or definite bruxism according to current consensus criteria, they remain a methodologically appropriate and widely accepted approach for large-scale population-based research when instrumental assessments are not feasible.

In this study, the presence of a single morning symptom (e.g., jaw fatigue or temporal pain) was considered sufficient to classify participants as having possible sleep bruxism (SB), in line with previously published self-report–based epidemiological protocols [2]. As symptom frequency or regularity was not assessed in the present study, such isolated symptoms may not necessarily reflect regular SB activity, and reliance on a one-symptom threshold may lead to moderate overestimation of prevalence, which should be considered when interpreting the reported prevalence estimates.

In accordance with international consensus guidelines, it should be noted that self-report data allow classification only into possible bruxism, whereas probable and definite bruxism require clinical examination and/or instrumental assessments such as polysomnography or electromyography, which remain the gold-standard diagnostic methods. As no clinical or instrumental assessments were performed in the present study, classification into probable or definite bruxism was not possible, and sole reliance on self-report may result in moderate overestimation of true bruxism prevalence [1].

#### 2.3.3. Fonseca Anamnestic Index (FAI)

TMD-related symptoms were assessed using the FAI, which screens for possible TMD and has been verified for validity and reliability in Turkish by Kaynak et al. [32,33]. It was developed to examine the prevalence and the estimate of the seriousness of TMD, based on signs and symptoms, with a high degree of diagnostic accuracy [34]. The FAI consists of 10 questions assessing head and TMJ pain, joint movement limitations, parafunctional habits, occlusal disturbances, and emotional stress. Responses were scored as Yes (10 points), Sometimes (5 points), or No (0 points). The total score ranges from 0 to 100 and provides a TMD severity classification according to the FAI: 0–15 = no TMD, 20–40 = mild TMD, 45–65 = moderate TMD, and 70–100 = severe TMD. The items are presented in Supplementary Table S1.

The psychometric properties of the FAI have been well supported by multiple validation studies. The Turkish adaptation demonstrated good internal consistency (Cronbach’s α = 0.805) and strong test–retest reliability, with intraclass correlation coefficients ranging between 0.739 and 0.897 [33]. In larger validation cohorts, the diagnostic accuracy of the FAI has also been confirmed: when evaluated against the DC/TMD criteria, it showed high discriminative performance (AUC ≈ 0.93–0.98), very high sensitivity (94–98%), and moderate-to-high specificity (~88%). These findings collectively reinforce the FAI as a reliable screening tool for identifying pain-related and intra-articular TMDs in both clinical and epidemiological contexts [35]. In the present sample, the internal consistency of the FAI was acceptable (Cronbach’s α = 0.758), indicating adequate reliability for use in this study.

#### 2.3.4. Dental Environment Stress (DES) Scale

The Dental Environment Stress (DES) scale is a dentistry-specific instrument developed to evaluate the stress factors encountered by dental students during their education. It was first introduced by Garbee et al. in 1980, and subsequent validity and reliability studies have been conducted in different countries [18]. In Türkiye, it was adapted into Turkish and its psychometric properties were confirmed by Ozyurtseven and Gungormus in 2021 [36]. The scale evaluates students’ sources of stress across multiple domains, including academic workload (course intensity, assignments, examinations), clinical practice (patient communication, clinical responsibilities, treatment procedures), and environmental or social factors (financial difficulties, living conditions, limited rest time). Each item is rated on a 5-point Likert scale (1 = not stressful at all to 5 = extremely stressful). Accordingly, the total DES score ranges from 31 to 155 with higher scores indicating greater perceived stress. The total score reflects the overall level of perceived stress. In the present study, we used a modified 31-item version of the Turkish DES questionnaire as described by Peker et al. [20]. The full list of DES items and their allocation to subscales are provided in Supplementary Table S2.

The psychometric properties of the Dental Environment Stress (DES) scale have been confirmed across multiple international validation studies. The Turkish adaptation demonstrated satisfactory construct validity and strong internal consistency (Cronbach’s α = 0.919), supporting its use in dental student populations [36]. Similar findings—high internal consistency and stable factor structures—have been reported in German and Romanian adaptations [37,38]. Collectively, these results indicate that the DES is a reliable and valid instrument for assessing perceived stress in dental education, and its use in the present study aligns with established international applications. In the present study, the DES demonstrated excellent internal consistency (Cronbach’s α = 0.964), confirming its reliability in the current sample.

### 2.4. Statistical Analysis

All statistical analyses were conducted using IBM SPSS Statistics (version 22.0; IBM Corp., Armonk, NY, USA). Continuous variables are reported as mean ± standard deviation, whereas categorical variables are presented as frequencies and percentages.

Data normality was evaluated using the Kolmogorov–Smirnov test. As the distributions deviated from normality, non-parametric statistical methods were applied. Accordingly, comparisons between the bruxism and non-bruxism groups for total FAI scores, total DES scores, and DES subscale scores were performed using the Mann–Whitney U test. Categorical variables were compared using the chi-square test. Associations between FAI and DES scores were assessed using Spearman’s rank correlation coefficient.

To complement *p*-values and provide information on the magnitude of observed effects, effect size estimates were calculated. For between-group comparisons of total FAI scores, total DES scores, and DES subscale scores, effect size r was derived from the standardized Mann–Whitney U test statistic (r = z/√N) and corresponding 95% confidence intervals were reported.

For item-level DES comparisons, standardized mean differences were additionally calculated to facilitate interpretation. Cohen’s d with Hedges’ g correction was used to account for unequal group sizes and potential small-sample bias, and 95% confidence intervals were provided. This supplementary analysis was included to enhance the interpretability of individual item-level effects.

For correlation analyses, Spearman’s rho coefficients were accompanied by 95% confidence intervals calculated using Fisher’s z transformation [39].

Effect sizes were interpreted in accordance with current methodological recommendations in dental research: for correlations, r = 0.20 (small), 0.40 (medium), and 0.70 (large); and for standardized mean differences, Cohen’s d = 0.10 (small), 0.40 (medium), and 0.90 (large) [40]. This approach enabled interpretation of both statistical significance and clinical relevance. A *p*-value < 0.05 was considered statistically significant.

## 3. Results

A total of 287 dental students (194 females, 93 males) with a mean age of 21.45 years participated in the study. Of these, 146 were preclinical students (1st, 2nd, and 3rd years) and 141 were clinical students (4th and 5th years) (Table 1).

Bruxism was identified in 76% of the participants (Table 1). The prevalence was significantly higher in female students than in male students (*p* = 0.003). Although the prevalence was highest among 5th-year students (86.1%), the difference across academic years did not reach statistical significance. (Table 1) Apart from gender, no significant differences were observed between bruxism and non-bruxism groups in terms of demographic variables.

Regarding the total score of the Fonseca Anamnestic Index (FAI), students with bruxism had significantly higher scores (*p* < 0.0001) compared to those without bruxism (Table 2). Similarly, the total and average score obtained from the Dental Environment Stress (DES) scale was also significantly higher in the bruxism group than in the non-bruxism group (*p* = 0.002) (Table 2). When DES subscales were examined, students with bruxism showed significantly higher stress scores in the domains of Academic Performance, Institutional Factors, Professional Future, Responsibilities with Patients, and Social Factors compared to non-bruxers (Table 2). Effect size analysis showed a large difference for FAI scores (r = 0.44), whereas the differences in total DES scores and DES subscales were of small magnitude (r = 0.13–0.19). This indicates that although stress levels were statistically higher in the bruxism group, the practical and clinical magnitude of these differences was limited.

Also, a positive correlation was observed between the total Fonseca and total DES scores (Table 3); this association was statistically significant in the bruxism group (r = 0.229, 95% CI: 0.10–0.35, *p* < 0.001), whereas no significant correlation was found in the non-bruxism group (r = 0.122, 95% CI: −0.12–0.35, *p* = 0.317). The magnitude of the observed association in the bruxism group corresponded to a small effect size, suggesting statistical but limited clinical relevance.

The comparison of individual Dental Environment Stress (DES) items between students with and without bruxism is presented in Table 4. To complement non-parametric group comparisons, standardized mean differences (Hedges’ g) with 95% confidence intervals were calculated at the item level to facilitate interpretation of effect magnitude.

Comparison of DES items between bruxism and non-bruxism groups revealed significant differences in several domains (Table 4). The bruxism group reported significantly higher scores for coursework load (Item 1), coursework difficulty (Item 3), examinations (Item 4), Attitudes and behaviors of academic staff and assistants (Item 7), fear of failing specialty examination (Item 13), fear of unemployment (Item 14), lack of relaxation time (Item 16), poor living conditions (Item 17), financial difficulties (Item 19), learning clinical procedures (Item 22), lack of confidence in clinical decision-making (Item 24), shortage of clinical time (Item 25), responsibility to find patients (Item 26), inconsistent feedback from academic staff (Item 27), comprehensive patient care responsibility (Item 28), and difficulty cooperating with patients (Item 29) (*p* < 0.05).

Standardized mean differences (Hedges’ g) indicated that the magnitude of these differences was generally small to moderate, with the largest effects observed for lack of relaxation time (Item 16; g = 0.51), inconsistent feedback among academic staff (Item 27; g = 0.50), examinations (Item 4; g = 0.49), and fear of failing the specialty examination (Item 13; g = 0.42). For the remaining significant items, effect sizes were predominantly small (g ≈ 0.25–0.38), suggesting limited but consistent differences between groups.

No significant differences were observed in the remaining DES items between the two groups. (*p* > 0.05).

When the contribution of each item to the total Dental Environment Stress (DES) score was examined separately, statistically significant positive correlations were observed between all items and the total DES score in both groups (*p* < 0.001; Table 5). In the non-bruxism group, correlation coefficients ranged from r = 0.565 to 0.915, whereas in the bruxism group they ranged from r = 0.538 to 0.793. These correlations corresponded predominantly to medium-to-large and large effect sizes in both groups. Ninety-five percent confidence intervals (95% CIs) were calculated for all correlation coefficients to reflect the precision of the effect size estimates (Table 5).

In both groups, items related to clinical processes—namely lack of confidence in clinical decision-making (Item 24), difficulty in learning clinical procedures (Item 22), completion of clinical requirements (Item 23), and shortage of clinical time (Item 25)—showed the strongest correlations with the total DES score, indicating that these factors were the most strongly associated with overall stress levels.

## 4. Discussion

Dental education is widely recognized as one of the most stressful training processes in the health sciences, demanding intensive theoretical knowledge, the development of clinical skills, patient management, and effective communication [13,41]. The simultaneous pressure to achieve academic success, gain clinical competence, and assume responsibility for patient care places dental students under substantially higher levels of psychosocial stress compared to many other health professional students [15]. Previous literature highlights that such elevated stress levels not only impair quality of life but may also represent a significant contributing factor in the development of bruxism [6,24]. Accordingly, the considerable stress burden observed in dental students may be associated with bruxism in this population. Nonetheless, much of the existing research has either relied on general stress scales or examined bruxism and TMD separately. To address this gap, the present study aimed to comprehensively investigate the interplay between education-specific stress, bruxism, and TMD-related symptoms. This was achieved by simultaneously assessing self-reported bruxism, TMD-related symptoms using the Fonseca Anamnesis Index (FAI), and dental education-specific stressors using the Dental Environmental Stress (DES) scale.

Bruxism prevalence (76%) observed in our cohort falls within the upper range of values reported for dental student populations internationally, a group consistently shown to exhibit higher rates than the general population (8–31.6%) and the global prevalence identified in meta-analyses (22%), largely due to the demanding nature of dental education [5,42]. Similarly, studies focusing on university populations have consistently reported higher rates, varying from 31% to 70% [25,43]. Among dental students specifically, prevalence has been reported across a broad spectrum, from 21.5% to 76%, which is likely attributable to differences in diagnostic criteria, assessment methods, cultural factors, and educational contexts [9,21,44,45,46,47]. Against this background, the considerably high prevalence identified in our study underscores that the demanding academic and clinical workload, coupled with the competitive structure of dental education in Türkiye, may be one of the contextual factors related to the elevated bruxism prevalence observed. In addition, the reliance on self-reported bruxism measures may have contributed to an overestimation of prevalence, and this methodological aspect should be considered when interpreting the relatively high rates observed in the present study.

Given this markedly elevated prevalence, the relatively smaller size of the non-bruxism group in our sample reflects the true distribution of the phenomenon within this population rather than a sampling imbalance. Similar patterns have been reported in studies where bruxism prevalence among dental students exceeds 60–70%, resulting in proportionally small non-bruxer subgroups. Accordingly, the group distribution observed in the present study represents an expected epidemiological outcome in a high-risk cohort exposed to substantial academic and psychosocial demands. In addition, all statistical analyses were performed using non-parametric tests appropriate for unequal group sizes, ensuring the robustness of the observed associations despite the asymmetry between groups.

According to the results, no significant differences were observed across grades; however, the prevalence of bruxism was significantly higher in female students than in males. The literature on gender differences remains inconclusive. Some studies suggest that bruxism is independent of gender and report no significant differences between men and women [42,44,46]. Conversely, numerous studies—consistent with our results—have found higher rates of bruxism among women [9,21,43,45,48,49]. This higher prevalence in females may be linked not only to psychosocial stressors but also to hormonal and neurobiological differences. Indeed, previous research shows that women tend to be more sensitive to stress, employ different coping strategies, and exhibit greater activation of the prefrontal cortex during stress responses [50,51].

Biologically, gender-related hormonal differences, particularly estrogen fluctuations, have been shown to affect stress-related neuroendocrine responses, pain perception, and muscle activity [52,53]. This may increase predisposition to stress-related parafunctional behaviors such as bruxism. Furthermore, women tend to adopt internalizing coping patterns; in these patterns, psychological stress is expressed through somatic symptoms rather than overt behavioral responses, which may facilitate the manifestation of stress through masticatory muscle activity [54].

These biopsychosocial mechanisms may explain why bruxism is more common in female students under high stress, supporting the view that its development is influenced not only by academic workload but also by gender-related factors [43]. Taken together, these biological and behavioral factors may contribute to the higher prevalence of bruxism observed among female dental students and emphasize the importance of considering gender-related stress responses when interpreting stress-related oral parafunctions.

While self-report methods are practical for assessing bruxism, they have significant limitations, particularly for sleep bruxism, such as low sensitivity and a tendency toward over-reporting. This indicates that the high prevalence observed in our study may partly reflect measurement-related bias. Comparative studies using PSG or EMG have shown poor agreement between self-report and objective measurements, and that self-report systematically overestimates the rates of bruxism confirmed by PSG [55]. Therefore, when interpreting the prevalence levels obtained, the methodological limitations of self-reporting should be taken into account [31]. The use of objective methods in the future will contribute to a more reliable determination of the true prevalence of bruxism and possible stress-related perceptual effects.

Students with bruxism showed higher FAI scores, indicating more pronounced TMD-related symptoms in this group and reinforcing the close association between bruxism and temporomandibular dysfunction previously reported in dental student populations [56,57]. Broader evidence similarly demonstrates increased TMD prevalence and higher FAI scores among individuals with bruxism, confirming a strong relationship between bruxism and TMD symptoms [11,22,44,58,59]. Mechanistically, repetitive masticatory muscle activity and excessive occlusal loading characteristic of bruxism have been proposed as potential mechanisms linked to microtrauma, inflammation, disc displacement, and degenerative joint changes, underscoring the importance of considering bruxism in both diagnostic evaluations and preventive management of TMD [60,61]. Notably, effect size analysis demonstrated a moderate-to-large magnitude for the difference in FAI scores between students with and without bruxism (r = 0.44), indicating that this association is not only statistically significant but also clinically meaningful. This magnitude suggests potential clinical relevance in terms of symptom burden.

In addition, the significantly higher total and mean DES scores, as well as higher scores across DES subscales, observed in the bruxism group in our study indicate that stress factors specific to dental education are consistently associated with bruxism. However, effect size analysis indicated that the differences observed in total DES scores and DES subscale scores were of small magnitude (r = 0.13–0.19). This suggests that although dental education–related stress was consistently higher among students with bruxism, the practical and clinical impact of these differences may be limited. Nevertheless, the systematic elevation across multiple DES domains—particularly those related to academic performance, institutional factors, professional future, and patient-related responsibilities—are consistent with cumulative stress burden rather than isolated stressors. This association is supported not only on a psychosocial level but has also been discussed in relation to biological mechanisms. Indeed, Fluerașu et al. demonstrated a positive correlation between sleep bruxism and salivary cortisol levels in young adults, and reported higher anxiety and stress levels, suggesting that both psychological and biological manifestations of stress contribute to the development of bruxism [62]. Similarly, a meta-analysis by Elani et al. showed that dental students experience sustained high stress throughout their education, particularly from academic and clinical processes, which negatively affects academic performance, physical health, and psychological well-being [15]. Consistently, previous research has also reported significant positive associations between perceived stress levels and bruxism in both the general population and student cohorts [7,9,25,42,45,48,58,63,64,65]. However, while most of these studies used general psychological scales (e.g., Perceived Stress Scale, DASS-21, Symptom Checklist-90), our study applied the DES scale, which directly measures stress sources specific to dental education. This allows for a more meaningful interpretation of findings within the dental education context. Accordingly, our study offers a valuable contribution to the literature by simultaneously examining the relationship between bruxism, TMD-related symptoms, and dental education-specific stress factors.

Although no standardized cut-off has been established for DES, scores of ≥2 have been suggested as indicative of increased stress [15]. In our study, students with bruxism reported higher DES scores compared with those without bruxism, and overall stress levels in the cohort appeared elevated relative to values commonly reported in previous studies. Given that DES scores in the literature typically fall within a moderate range, the higher levels observed here suggest that the academic and clinical demands of the local educational context may impose an unusually heavy stress burden on students [13,15,66,67,68,69,70,71]. These elevated values highlight that our cohort represents a particularly high-stress profile, underscoring the widespread and intense stress burden experienced by dental students.

In line with our findings, a significant positive association was observed between stress levels and TMD-related symptoms among students with bruxism. This pattern was not evident in those without bruxism, indicating that the co-occurrence of higher stress levels and TMD-related symptoms was more frequently observed in students with bruxism. Given the small effect size, this association should be interpreted cautiously and does not imply a directional or causal relationship. Rather, these findings highlight the multidimensional nature of bruxism, in which psychological stress and TMD-related symptoms tend to cluster. Consistent with this, previous studies have reported that students experiencing higher stress levels more frequently report TMD signs and symptoms [8,72,73].

Indeed, bruxism has been reported to be associated with muscle hyperactivity, masticatory muscle pain, joint sensitivity, and functional limitations [74,75,76]. These findings are in line with our results, further supporting the clinical relevance of the co-occurrence pattern between stress, bruxism, and TMD-related symptoms. Meta-analyses conducted in recent years also reinforce this association. Zieliński et al. reported a global co-occurrence rate of bruxism and TMD of 17%, with substantial intercontinental variation as North America 70%, South America 24%, Europe 14%, Asia 9% [77]. In addition, Mortazavi et al. found that bruxism increases the risk of TMD by more than two-fold [22]. Taken together, these findings, consistent with international data, highlight that bruxism is not merely an individual habit but rather a multidimensional clinical condition directly linked to temporomandibular health.

Although stress emerged as a significant contributing factor in our study, bruxism is known to be a multifactorial condition. In addition to psychosocial stress, poor sleep quality, high caffeine consumption or other stimulants, alcohol use, psychotropic medications, non-stress-related parafunctional habits, and certain occlusal or anatomical features may also influence the risk of bruxism. These variables were not evaluated in the present study and may partly account for individual variability in bruxism expression. Taking these additional factors into account will allow for a more balanced and comprehensive interpretation of the relationships identified. However, the focus on education-related stress remains justified given the study’s aims and the specific characteristics of the dental student population.

To further clarify which specific stressors differentiate students with and without bruxism, individual DES items were compared between groups (Table 4). In addition to statistical significance, effect size estimates were examined to evaluate the practical magnitude of these differences. Across the DES scale, examinations, fear of failure, anxiety about specialization examinations, concerns about unemployment and limited rest time emerged as the most prominent stressors in our cohort, echoing previous work that highlights workload and assessments as major contributors to dental student stress [15,18,66]. Consistent with earlier reports, grades, fear of failing a course or year, pressure to complete clinical requirements, lack of time for relaxation and difficulties in learning clinical procedures have repeatedly been identified as among the most stressful aspects of dental education [13,14,15,16,20,66,67,70,71]. In our sample, students with bruxism perceived these academic and clinical demands more intensely—particularly heavy workload, performance expectations, restricted clinical time, uncertainty in clinical decision-making and inconsistent feedback from staff—supporting evidence that specific academic and clinical pressures heighten psychological burden during training [15,20,66,68,69,78]. However, effect size analysis indicated that although these differences were statistically significant, their magnitude was generally small to moderate. Most DES items demonstrated small effect sizes, suggesting limited clinical relevance, whereas stressors related to insufficient relaxation time, examination pressure, fear of professional failure, and inconsistent academic feedback showed comparatively larger effects. This pattern indicates that while stress is broadly elevated among students with bruxism, only specific academic and clinical stressors may contribute meaningfully to the observed differences. Beyond these psychosocial pathways, stress-related physiological changes have been reported to correlate with increased masticatory muscle activity, which has also been observed in individuals with bruxism [43,79,80].

Clinical evidence further suggests that chronic stress has been associated with alterations in mechanisms controlling involuntary muscle activity by damaging the hippocampus and disrupting mesocortical dopaminergic pathways, while amygdala activation has been linked with rhythmic jaw activity in previous reports [81]. Collectively, these findings support the notion that the academic, clinical, and environmental stressors captured by the DES scale have been associated with bruxism in both psychological and biological domains.

In addition to academic and clinical stress, previous studies have highlighted the role of poor living conditions and financial difficulties in the development of bruxism, consistent with our findings [43,82]. Additionally, students living with their families have been reported to experience lower stress levels compared to those in dormitories or shared accommodation [83]. These findings reveal that environmental conditions outside the educational programme also affect students’ stress levels and parafunctional behaviors. Furthermore, the higher levels of post-graduation unemployment concerns and anxiety about failing the specialization exam among students with bruxism in our study likely reflect the economic challenges and employment limitations currently faced in Türkiye. In the Turkish context, the increasing number of dental graduates, limited employment opportunities, and intense competition in the specialization exam further exacerbate uncertainty about professional futures. Our findings indicate that the stress associated with such concerns may be related to increased bruxism reporting.

While our findings are generally consistent with the main stress factors reported in the literature, the most striking difference between students with and without bruxism emerged in the item related to inconsistent feedback from academic staff. When combined with other clinical stressors more frequently reported in the bruxism group, this inconsistency may undermine students’ confidence in their own clinical decisions, negatively affect perceptions of academic competence, and further elevate stress. This original finding highlights that academic consistency in the educational process is critical for students’ stress management and professional self-confidence. In the context of clinical dental education, students are required to make irreversible treatment-related decisions under supervision, often involving real patients. In such settings, inconsistent or contradictory academic feedback may increase uncertainty regarding clinical adequacy and heighten performance anxiety. This uncertainty may be particularly challenging for students already exposed to high academic and clinical demands, potentially amplifying stress-related responses. However, given the cross-sectional design of the present study, these associations should be interpreted as relational rather than causal. Therefore, ensuring consistency in feedback may not only reduce students’ psychological burden but also facilitate the healthier development of clinical decision-making skills and professional competence.

Previous studies have suggested that stress levels and related outcomes may evolve across academic years in dental education. Polychronopoulou et al. reported that preclinical students experience higher stress related to coursework load and examinations, whereas clinical-year students are more affected by patient treatment responsibilities and concerns regarding their professional future [84]. Similarly, Acharya et al. demonstrated that first-year students exhibit elevated stress levels due to unfamiliarity with the academic environment and adaptation difficulties [85]. In a cross-sectional study conducted in Brazil, Sangiorgio et al. found that fourth- and fifth-year students generally reported lower overall stress levels, except for items related to professional insecurity and lack of self-confidence [86]. Although no statistically significant differences across academic years were observed in the present study and no further year-based analyses were conducted, existing evidence suggests that stressors vary across stages of dental education, underscoring the need for future longitudinal and subgroup-based studies to clarify their potential impact on bruxism and to guide year-specific interventions.

When the relationships between individual DES items and overall stress were examined, all items were found to contribute meaningfully to students’ stress levels. Across both groups, the most influential stressors were those related to clinical processes, including uncertainty in clinical decision-making, difficulty learning clinical procedures, pressure to complete clinical requirements, and limited clinical time. These findings underscore that clinical demands play a central role in shaping students’ psychological burden. Regardless of the presence of bruxism, it is evident that the clinical learning environment and the development of clinical competence substantially influence overall stress levels.

Studies evaluating the contribution of each DES items to total stress through item–total correlation analyses remain limited. Al-Moosawi et al. (2020) reported significant correlations between DES items and total stress scores but neither investigated bruxism nor reported coefficients as high as those found in our study [87]. However, our study analysed item correlations separately in both the bruxism and non-bruxism groups and demonstrated in detail the contribution of clinical process-specific stress factors to overall stress levels. In this respect, it provides a rare and valuable contribution to the existing literature.

Although this study was conducted in Türkiye, similar challenges—such as high-stakes examinations, clinical time shortages, and lack of time for relaxation—have been reported in dental schools across Europe, Asia, and the Middle East [14,15,43,71]. This indicates that the stress–bruxism–TMD cycle is a global issue with implications for both educational quality and healthcare workforce sustainability. Addressing these factors institutionally could enhance student well-being locally and inform international best practices in dental training.

The remarkably high prevalence of bruxism (76%) observed in our cohort confirms that this condition is not a simple parafunctional habit but a multidimensional condition frequently reported alongside academic and clinical stressors, closely linked with temporomandibular disorders. Elevated FAI and DES scores, and their positive correlation, highlight how education-related stress was linked to higher TMD-related symptom levels through both psychosocial and biological pathways.

Collectively, these results demonstrate that the demanding workload of dental education is associated with reduced well-being among students, parafunctional behaviors, and temporomandibular health. Stress-related parafunctional disorders in dental students should therefore be recognized not only as an academic concern but also as a public health issue. As future members of the oral healthcare workforce, students experiencing unaddressed stress may show reduced clinical performance, higher risk of musculoskeletal problems, and compromised long-term professional capacity, ultimately affecting patient care. These findings emphasize the importance of addressing stress and bruxism during training to support individual well-being and potentially contribute to the resilience of the healthcare workforce.

In recent years, curriculum-based, multi-component interventions designed to support students’ psychological and physiological well-being have become increasingly common in health and dental education. Many institutions have incorporated stress-management modules, resilience training, mindfulness-based practices, and bio-behavioral approaches either within formal curricula or as complementary educational activities. Previous studies consistently report reductions in perceived stress, examination anxiety, emotional burnout, and general anxiety levels, alongside improvements in coping capacity, self-awareness, academic functioning, and resilience [88,89,90]. Furthermore, mindfulness-based programs have additionally been associated with reduced psychological distress, modulation of stress-related biomarkers, and improvements in learning and clinical performance among both students and clinicians [91,92,93,94].

By contrast, direct evidence demonstrating a measurable reduction in bruxism prevalence following educational wellness programs remains limited. Nevertheless, a substantial body of clinical research indicates that cognitive-behavioral therapy, bio-behavioral interventions, mindfulness-based pain approaches, and postural-behavioral modification protocols applied in TMD populations result in meaningful improvements in pain intensity, muscle tension, and functional outcomes [95,96,97,98]. These findings provide a reasonable biological and behavioral rationale suggesting that stress reduction strategies may influence the mechanisms associated with bruxism and TMD.

Accordingly, integrating structured stress-reduction strategies, such as mindfulness modules, stress management training, counseling systems, improvements in academic feedback processes, and resilience-building programs, into dental education may be considered to support student well-being and potentially reduce stress-related symptom burden. However, longitudinal and intervention-based studies are still required to quantitatively determine the extent to which such programs influence bruxism and TMD outcomes.

### 4.1. Practical Implications

- Inconsistent feedback from academic staff emerged as the most prominent modifiable stressor; implementing structured and consistent feedback systems may help reduce students’ stress levels.
- Counselling services and structured stress-, time-, and workload-management training may support student well-being and could be associated with a lower risk of stress-related bruxism.
- Strengthening institutional support mechanisms—particularly those addressing academic workload, clinical uncertainty, and feedback quality—may enhance professional resilience during dental training.
- These findings provide a foundation for evidence-based curricular adjustments aimed at fostering healthier learning environments and promoting sustainable professional development.
- Further multicentre and longitudinal research is required to guide the development and evaluation of effective preventive interventions.

### 4.2. Limitations

This study has several limitations. Bruxism was assessed exclusively through self-reports, without clinical examinations or objective diagnostic methods (e.g., EMG, polysomnography). Consequently, participants could only be classified as having possible bruxism, and differentiation between sleep and awake bruxism was not feasible. While this approach is commonly adopted in large-scale epidemiological studies, it inherently limits diagnostic accuracy and may contribute to imprecise prevalence estimates.

In addition, potential variations in the stress–bruxism relationship across different academic years were not explored, despite evidence that stress exposure and stressors may change throughout dental education. Although the sample size exceeded the minimum required by power analysis, larger and more balanced cohorts would be necessary to enable robust subgroup analyses by gender and academic level. Furthermore, individual differences in anxiety, coping strategies, and stress perception—factors not evaluated in the present study—may have influenced the observed associations.

Finally, the cross-sectional design precludes causal inferences. Future research should therefore employ multi-center and cross-cultural cohorts, incorporate objective diagnostic methods (e.g., EMG, PSG), and adopt longitudinal designs to clarify causality. Comparative studies across different dental education systems would also provide valuable insights into how curricular structures and cultural contexts shape the stress–bruxism–TMD relationship.

## 5. Conclusions

- The prevalence of bruxism among dental students was very high (76%) and significantly higher in females than in males.
- Students with bruxism exhibited higher DES and FAI scores, and the positive correlation between these scales indicates a significant association with both TMD-related symptoms and education-related stress.
- Academic, clinical, and environmental stressors—including inconsistent academic feedback, examinations, insufficient rest time, concerns about professional future, patient-related responsibilities, difficulty finding patients, insecurity in clinical decision-making, and financial difficulties—were more pronounced among students with bruxism.
- These findings demonstrate that not only the intensity of stress but also the type of stressor plays a critical role in the manifestation of bruxism.
- Overall, bruxism appears to be a widespread stress-related condition among dental students, closely linked with TMD-related symptoms and education-specific stressors.
- Beyond its academic significance, the association between stress, bruxism, and TMD-related symptoms observed in this study has important clinical implications. Persistent stress-related oral parafunctions and TMD symptoms may impair concentration, manual dexterity, and clinical performance, potentially affecting the quality of patient care. Addressing stress during dental education is therefore essential not only for student well-being but also for safeguarding the long-term health and professional effectiveness of future dental practitioners.

## Figures and Tables

**Table 1:** Sociodemographic characteristics of students with and without bruxism and results of the Chi-square test.

		Non-Bruxism Group (n = 69)		Bruxism Group (n = 218)		Total		Chi-Square Test
		n	%	n	%	n	%	*p*
Years of Study	1st grade	11	28.2	28	71.8	39	100.0	0.237
	2nd grade	9	29.0	22	71.0	31	100.0	
	3rd grade	20	26.3	56	73.7	76	100.0	
	4th grade	19	27.5	50	72.5	69	100.0	
	5th grade	10	13.9	62	86.1	72	100.0	
Gender	Female	36	18.6	158	81.4	194	100.0	0.003 *
	Male	33	35.5	60	64.5	93	100.0	
Income Level	Low	16	22.9	54	77.1	70	100.0	0.109
	Middle	46	22.8	156	77.2	202	100.0	
	High	7	46.7	8	53.3	15	100.0	
Smoking status	Non-smoker	50	24.4	155	75.6	205	100.0	0.948
	Smoker	19	23.2	63	76.8	82	100.0	
Alcohol consumption	Non-drinker	44	24.2	138	75.8	182	100.0	0.948
	Drinker	24	23.1	80	76.9	104	100.0	
Chewing side preference	Left	1	5.9	16	94.1	17	100.0	0.086
	Right	6	16.7	30	83.3	36	100.0	
	Bilateral	62	26.5	172	73.5	234	100.0	
	Total	69	24.0	218	76.0	287	100.0	

n: number of participants; values are presented as n (%); *p*-values were calculated using the chi-square test; *: *p* < 0.05.

**Table 2:** Comparison of FAI, DES total scores, and DES subscale scores between students with and without bruxism using the Mann–Whitney U test.

	Non-Bruxism Group (n = 69)	Bruxism Group (n = 218)	z	*p*	Effect Size (r)	CI 95%
	X ± SD	X ± SD				
**Fonseca Anamnestic** **Index (FAI)**	19.71 ± 17.08	41.81 ± 20.32	−7.40	0.0001 *	0.44	0.34–0.53
**DES—mean score**	3.01 ± 0.88	3.34 ± 0.84	−3.09	0.002 *	0.18	0.07–0.29
**DES—total score**	93.30 ± 27.25	103.57 ± 26.21	−3.09	0.002 *	0.18	0.07–0.29
**DES—subscale score**						
Academic Performance	30.90 ± 8.63	33.65 ± 8.84	−2.48	0.014 *	0.15	0.03–0.26
Institutional Factors	15.77 ± 4.91	17.60 ± 4.67	−2.74	0.006 *	0.16	0.05–0.27
Professional Future	9.78 ± 3.04	11.09 ± 3.03	−3.21	0.001 *	0.19	0.08–0.30
Responsibilities with Patients	20.62 ± 6.92	23.33 ± 6.88	−3.10	0.002 *	0.18	0.07–0.29
Social Factors	16.23 ± 6.21	17.90 ± 6.21	−2.18	0.029 *	0.13	0.01–0.24

n: number of participants; values are presented as mean ± standard deviation (X ± SD); z: standardized test statistic from the Mann–Whitney U test; r: effect size calculated as z/√N; 95% CI: 95% confidence interval; *: *p* < 0.05.

**Table 3:** Spearman’s Correlation Between Fonseca Anamnestic Index and Total Dental Environment Stress (DES) Scores in Bruxism and Non-Bruxism Groups.

Group	Variables	r	*p*	95% CI
**Non-Bruxism Group**	Fonseca total score—Total DES score	0.122	0.317	−0.12–0.35
**Bruxism Group**	Fonseca total score—Total DES score	0.229	<0.001 *	0.10–0.35

r: Spearman’s correlation coefficient; 95% CI: 95% confidence interval; *: *p* < 0.05.

**Table 4:** Comparison of individual Dental Environment Stress (DES) items between students with and without bruxism using Mann–Whitney U test and effect size estimates (Hedges’ g).

DES Items (Shortened)		Mann–Whitney U Test				
		Non-Bruxism Group (n = 69)	Bruxism Group (n = 218)	*p*	Hedges’ g	95% CI
		X ± SD	X ± SD			
1.	Coursework load	3.2 ± 1.0	3.6 ± 1.1	0.014 *	0.370	0.10–0.64
2.	Manual dexterity difficulty	3.2 ± 1.1	3.3 ± 1.2	0.576	0.085	−0.19–0.35
3.	Coursework difficulty	3.1 ± 0.9	3.5 ± 1.1	0.008 *	0.378	0.11–0.65
4.	Examinations	3.6 ± 1.1	4.1 ± 1.0	0.001 *	0.487	0.21–0.76
5.	Learning precision skills	3.0 ± 1.1	3.2 ± 1.2	0.221	0.169	−0.10–0.44
6.	Staff atmosphere	3.1 ± 1.1	3.4 ± 1.3	0.114	0.241	−0.03–0.51
7.	Staff/assistant attitudes	3.2 ± 1.2	3.6 ± 1.1	0.010 *	0.376	0.10–0.65
8.	Lack of self-confidence	2.9 ± 1.2	3.2 ± 1.3	0.091	0.236	−0.04–0.51
9.	Peer competition (grades)	2.4 ± 1.1	2.4 ± 1.3	0.856	0.001	−0.27–0.27
10.	Infection risk (HBV/HCV/HIV)	2.9 ± 1.2	3.2 ± 1.3	0.109	0.220	−0.05–0.49
11.	Faculty rules/regulations	3.3 ± 1.1	3.5 ± 1.3	0.334	0.164	−0.11–0.44
12.	Fear of failing year	3.7 ± 1.2	4.0 ± 1.2	0.088	0.252	−0.02–0.52
13.	Fear of failing specialty exam	3.5 ± 1.1	4.0 ± 1.2	0.002 *	0.424	0.15–0.70
14.	Fear of unemployment	3.4 ± 1.2	3.9 ± 1.2	0.002 *	0.416	0.14–0.69
15.	Fear of facing parents after failure	3.3 ± 1.4	3.6 ± 1.4	0.085	0.213	−0.06–0.49
16.	Lack of relaxation time	3.3 ± 1.1	3.9 ± 1.2	0.001 *	0.508	0.24–0.78
17.	Poor living conditions	2.8 ± 1.3	3.2 ± 1.5	0.031 *	0.289	0.02–0.56
18.	Opposite-sex relationships	2.5 ± 1.3	2.8 ± 1.4	0.201	0.218	−0.05–0.49
19.	Financial difficulties	2.7 ± 1.2	3.2 ± 1.3	0.007 *	0.383	0.11–0.66
20.	Delay of marriage	2.3 ± 1.4	2.2 ± 1.4	0.598	−0.071	−0.34–0.20
21.	Physical health problems	2.7 ± 1.2	3.0 ± 1.3	0.134	0.195	−0.08–0.47
22.	Learning clinical procedures	2.9 ± 1.2	3.2 ± 1.2	0.029 *	0.250	−0.02–0.52
23.	Clinical requirements	2.7 ± 1.2	2.9 ± 1.3	0.200	0.160	−0.11–0.43
24.	Lack of confidence in deciding	2.9 ± 1.2	3.3 ± 1.3	0.031 *	0.318	0.05–0.59
25.	Shortage of clinical time	2.9 ± 1.2	3.3 ± 1.2	0.011 *	0.322	0.05–0.59
26.	Responsibility to find patients	3.2 ± 1.3	3.7 ± 1.3	0.005 *	0.383	0.11–0.66
27.	Differences among academic staff	2.9 ± 1.2	3.5 ± 1.2	<0.001 *	0.499	0.23–0.77
28.	Patient care responsibility	3.2 ± 1.1	3.6 ± 1.2	0.009 *	0.353	0.08–0.63
29.	Difficulty cooperating with patients	2.6 ± 1.2	3.0 ± 1.2	0.024 *	0.318	0.05–0.59
30.	Patients late or absent	2.8 ± 1.2	3.2 ± 1.3	0.054	0.278	0.01–0.55
31.	Treating patients with poor hygiene	3.0 ± 1.1	3.2 ± 1.3	0.443	0.164	−0.11–0.44

n: number of participants; X ± SD: mean ± standard deviation; g: Hedges’ g (standardized mean difference); 95% CI: 95% confidence interval; effect size interpretation: small ≈ 0.10, medium ≈ 0.40, large ≈ 0.90; *: *p* < 0.05.

**Table 5:** Spearman’s correlation coefficients between individual Dental Environment Stress (DES) items in bruxism and non-bruxism groups.

DES Items (Shortened)		Spearman’s Correlation Test					
		Non-Bruxism Group (n = 69)			Bruxism Group (n = 218)		
		r	*p*	95% CI	r	*p*	95% CI
1.	Coursework load	0.595 *	<0.001	0.42–0.73	0.677 *	<0.001	0.60–0.74
2.	Manual dexterity difficulty	0.651 *	<0.001	0.49–0.77	0.654 *	<0.001	0.57–0.72
3.	Coursework difficulty	0.719 *	<0.001	0.58–0.82	0.733 *	<0.001	0.67–0.79
4.	Examinations	0.690 *	<0.001	0.54–0.80	0.554 *	<0.001	0.46–0.64
5.	Learning precision skills	0.738 *	<0.001	0.61–0.83	0.724 *	<0.001	0.65–0.78
6.	Staff atmosphere	0.829 *	<0.001	0.74–0.89	0.702 *	<0.001	0.63–0.77
7.	Staff/assistant attitudes	0.715 *	<0.001	0.58–0.81	0.658 *	<0.001	0.58–0.73
8.	Lack of self-confidence	0.794 *	<0.001	0.69–0.87	0.698 *	<0.001	0.62–0.76
9.	Peer competition (grades)	0.675 *	<0.001	0.52–0.79	0.646 *	<0.001	0.56–0.72
10.	Infection risk (HBV/HCV/HIV)	0.719 *	<0.001	0.58–0.82	0.640 *	<0.001	0.55–0.71
11.	Faculty rules/regulations	0.782 *	<0.001	0.67–0.86	0.749 *	<0.001	0.69–0.80
12.	Fear of failing year	0.640 *	<0.001	0.48–0.76	0.645 *	<0.001	0.56–0.72
13.	Fear of failing specialty exam	0.706 *	<0.001	0.56–0.81	0.627 *	<0.001	0.54–0.70
14.	Fear of unemployment	0.768 *	<0.001	0.65–0.85	0.668 *	<0.001	0.59–0.73
15.	Fear of facing parents after failure	0.650 *	<0.001	0.49–0.77	0.549 *	<0.001	0.45–0.64
16.	Lack of relaxation time	0.714 *	<0.001	0.57–0.81	0.651 *	<0.001	0.57–0.72
17.	Poor living conditions	0.824 *	<0.001	0.73–0.89	0.591 *	<0.001	0.50–0.67
18.	Opposite-sex relationships	0.701 *	<0.001	0.56–0.80	0.625 *	<0.001	0.53–0.70
19.	Financial difficulties	0.690 *	<0.001	0.54–0.80	0.638 *	<0.001	0.55–0.71
20.	Delay of marriage	0.565 *	<0.001	0.38–0.71	0.538 *	<0.001	0.44–0.63
21.	Physical health problems	0.789 *	<0.001	0.68–0.86	0.704 *	<0.001	0.63–0.77
22.	Learning clinical procedures	0.881 *	<0.001	0.81–0.93	0.793 *	<0.001	0.74–0.84
23.	Clinical requirements	0.868 *	<0.001	0.80–0.92	0.747 *	<0.001	0.68–0.80
24.	Lack of confidence in deciding	0.915 *	<0.001	0.87–0.95	0.769 *	<0.001	0.71–0.82
25.	Shortage of clinical time	0.850 *	<0.001	0.77–0.91	0.757 *	<0.001	0.70–0.81
26.	Responsibility to find patients	0.790 *	<0.001	0.68–0.87	0.739 *	<0.001	0.67–0.79
27.	Differences among academic staff	0.850 *	<0.001	0.77–0.92	0.726 *	<0.001	0.66–0.78
28.	Patient care responsibility	0.826 *	<0.001	0.73–0.89	0.720 *	<0.001	0.65–0.78
29.	Difficulty cooperating with patients	0.735 *	<0.001	0.60–0.83	0.724 *	<0.001	0.65–0.78
30.	Patients late or absent	0.787 *	<0.001	0.68–0.87	0.710 *	<0.001	0.64–0.78
31.	Treating patients with poor hygiene	0.571 *	<0.001	0.39–0.71	0.638 *	<0.001	0.55–0.71

n: number of participants; r: Spearman’s correlation coefficient; 95% CI: 95% confidence interval; *: *p* < 0.05.

## Data Availability

The data presented in this study are available upon request from the corresponding author due to ethical restrictions and the presence of sensitive participant information. Access to the dataset can be provided upon reasonable request, provided that it complies with institutional ethics requirements.

## Supplementary Materials

The following supporting information can be downloaded at https://www.mdpi.com/article/10.3390/healthcare14010072/s1. Table S1: Fonseca Anamnestic Index (FAI); Table S2: Modified Dental Environment Stress (DES) Scale: Item Classification by Subscales.

## Abbreviations

The following abbreviations are used in this manuscript:

TMD	Temporomandibular Disorders
TMJ	Temporomandibular Joint
DES	Dental Environment Stress
FAI	Fonseca Anamnestic Index
SB	Sleep Bruxism
AB	Awake Bruxism
