# Supplementary material for: Education-Related Stress and Its Behavioral and Somatic Manifestations Among Dental Students: A Cross-Sectional Analysis of Bruxism and Temporomandibular Symptoms
Source: Healthcare (Basel). 2025 Dec 27;14(1):72. doi: 10.3390/healthcare14010072 (PMC12785466; doi:10.3390/healthcare14010072)
Supplement: Supplementary file 1 [file healthcare-14-00072-s001.zip › Supplementary Table S1.pdf]

Supplementary Table S1. (FAI)

The following questionnaire was provided to participants in this study.

The Fonseca Anamnestic Index (FAI) is a 10-item questionnaire used to evaluate temporomandibular dysfunction. Each question is answered as "Yes" (10 points), "Sometimes" (5 points), or "No" (0 points). The sum of scores determines the severity of TMD.

**Fonseca Anamnestic Index**	**Yes**	**Sometimes**	**No**
Do you have difficulty opening your mouth widely?			
Do you have difficulty moving your lower jaw left or right?			
Do you feel muscle fatigue/pain when chewing?			
Do you often have headaches?			
Do you have neck pain or neck stiffness?			
Do you have pain in your ear or jaw joint?			
Do you hear any clicking sounds from the jaw joint when chewing or opening your mouth?			
Do you have a habit of clenching or grinding your teeth?			
Do you feel that your teeth do not come together well?			
Do you think you are a nervous (irritable) person?			

Classification of TMD severity: 0–15 points = No TMD; 20–40 points = Mild TMD; 45–65 points = Moderate TMD; 70–100 points = Severe TMD
