# Supplementary material for: Education-Related Stress and Its Behavioral and Somatic Manifestations Among Dental Students: A Cross-Sectional Analysis of Bruxism and Temporomandibular Symptoms
Source: Healthcare (Basel). 2025 Dec 27;14(1):72. doi: 10.3390/healthcare14010072 (PMC12785466; doi:10.3390/healthcare14010072)
Supplement: Supplementary file 1 [file healthcare-14-00072-s001.zip › Supplementary Table S2.pdf]

Supplementary Table S2. Modified Dental Environment Stress (DES) Scale: Item Classification by Subscales

The Modified Dental Environment Stress (DES) Scale is a self-report questionnaire designed to assess sources of stress among dental students. The scale consists of 31 items rated on a 5-point Likert scale (1 = not stressful at all; 5 = extremely stressful), with higher scores indicating greater perceived stress in the dental educational environment.

For transparency and consistency with the statistical analyses presented in the main manuscript, DES items are presented below grouped according to their predefined subscales: Academic Performance, Institutional Factors, Professional Future, Responsibilities with Patients, and Social Factors.

**DES ITEMS (Stressors) According to Subscale**
**Academic Performance**
Item 1. The amount of assigned coursework in practical classes during the academic year
Item 2. Difficulty experienced in procedures requiring manual dexterity
Item 3. Difficulty of coursework
Item 4. Examinations
Item 5. Difficulty in learning precision manual skills required for clinical and laboratory work
Item 9. Competition with peers for grades
Item 12. Fear of failing the year
Item 22. Difficulty in learning clinical procedures
Item 23. Completion of clinical requirements (e.g., dress code, entry-exit times, sterilization protocols, etc.)
Item 24. Lack of confidence in clinical decision making
**Institutional Factors**
Item 6. Atmosphere created by preclinical and/or clinical staff
Item 7. Attitudes and behaviors of academic staff and assistants
Item 11. Rules and regulations of the dental faculty
Item 16. Lack of time for relaxation

Item 25. Shortage of allocated clinical time

Professional Future

Item 8. Lack of confidence to be a successful dentist

Item 13. Fear of failing the Dental Specialization Examination / Fear of not being accepted into postgraduate programs

Item 14. Fear of unemployment after graduation

Responsibilities with Patients

Item 10. Risk of infectious diseases (HBV, HCV, HIV etc.)

Item 26. Responsibility for finding suitable patients

Item 27. Differences of opinion among consulted academic staff during patient evaluation (diagnosis or treatment)

Item 28. Responsibility for comprehensive patient care

Item 29. Difficulty of cooperation with patients

Item 30. Patients being late or not showing for appointments

Item 31. Working on patients with poor oral hygiene

Social Factors

Item 15. Fear of facing parents after failure

Item 17. Uncomfortable living conditions

Item 18. Relationship between members of the opposite sex

Item 19. Financial difficulties

Item 20. Forced postponement of marriage or engagement

Item 21. Personal physical health
